# Efficacy of cannabinoids for the prophylaxis of chemotherapy-induced nausea and vomiting—a systematic review and meta-analysis

**DOI:** 10.1007/s00520-025-09251-w

**Published:** 2025-02-14

**Authors:** Ronald Chow, Anna Basu, Jagdeep Kaur, David Hui, James Im, Elizabeth Prsic, Gabriel Boldt, Michael Lock, Lawson Eng, Terry L. Ng, Camilla Zimmermann, Florian Scotte

**Affiliations:** 1 Princess Margaret Cancer Centre, Temerty Faculty of Medicine, University of Toronto, Toronto, ON Canada; 2 Centre for Evidence-Based Medicine, University of Oxford, Oxford, UK; 3 Verspeeten Family Cancer Centre, Schulich School of Medicine & Dentistry, University of Western Ontario, London, ON Canada; 4 The University of Texas MD Anderson Cancer Center, Houston, TX USA; 5 Faculty of Medicine and Dentistry, University of Alberta, Edmonton, AB Canada; 6 Yale School of Medicine, Yale University, New Haven, CT USA; 7 Faculty of Medicine, The Ottawa Hospital Cancer Centre, University of Ottawa, Ottawa, ON Canada; 8 Gustave Roussy, Université Paris-Saclay Faculté de Médecine, Université Paris-Saclay, Paris, France

**Keywords:** Cancer, Chemotherapy-induced nausea and vomiting, Antiemetic, Cannabinoid

## Abstract

**Background:**

Cannabinoids have potential efficacy as prophylaxis for chemotherapy-induced nausea and vomiting (CINV), but no recent meta-analysis has reported on their relative efficacy compared to other antiemetics. The aim of this meta-analysis is to examine the relative efficacy of cannabinoids for prophylaxis of CINV.

**Methods:**

A literature search was conducted in OVID Medline, EMBASE, and Cochrane Central Register of Controlled Trials from inception up until March 2024. Articles were included if they reported on complete response, no nausea, no vomiting or no use of rescue medications, and were randomized controlled trials with cannabinoids in one arm. Meta-analysis was conducted for each endpoint and for a composite endpoint amalgamating existing endpoints. Subgroup analyses by medication used in control arm and by study design were conducted. Cumulative and leave-one-out analysis was also conducted. Type I error was set at 0.05.

**Results:**

A total of 26 studies were included in this meta-analysis, of which 23 were published before the 2000s. Nearly half of the included studies had some concern for bias. Cannabinoid had superior overall CINV control compared to placebo (RR 2.65, 95% CI 1.70–4.12, *I*^2^ = 0.00%). However, there was no difference between cannabinoid and active treatment alternatives (most using dated single-agent regimens) for any outcomes. A recent phase II/III trial demonstrated superior efficacy of THC:CBD for secondary prevention of CINV when used as adjunctive therapy alongside modern antiemetic regimens, albeit mostly without olanzapine.

**Conclusions:**

There is scant evidence for efficacy of cannabinoids for CINV in the era of triple and quadruple antiemetics. Although THC:CBD showed promised in a recent trial, further trials should examine its safety and efficacy in the context of regimens containing olanzapine.

## Introduction

Chemotherapy-induced nausea and vomiting (CINV) is a common and debilitating adverse event of chemotherapy [1, 2], with a prevalence rate of over 50% among patients treated with highly emetogenic chemotherapy and over 40% among patients treated with moderately emetogenic chemotherapy [3]. If left untreated, it can lead to poor quality of life, lack of adherence to antineoplastic treatment, treatment termination, and poor oncologic outcomes [4, 5].

There has been extensive research on the pathophysiology of CINV. Acute CINV, which has been defined as CINV occurring 0–24 h after initiation of chemotherapy, is mediated predominantly by the peripheral pathway, while delayed CINV, defined as CINV occurring 24–120 h after initiation of chemotherapy, is mediated mainly by the central pathway [2, 6]. Prophylactic antiemetics developed to target these pathways have drastically reduced the incidence of CINV [7–12], but not to zero, especially for nausea.

Cannabinoids are being researched as well, for their potential effects on CINV [13]. There have been several systematic reviews reporting on the relative efficacy of cannabinoids compared to other antiemetics [14]. A recent meta-analysis in 2020 [15] found limited number of RCTs, many of which compared cannabinoids with dated treatment regimens for CINV that were no longer standard of practice. Since then, studies have been published comparing cannabinoids with newer regimens. The aim of this review is to examine the relative efficacy of cannabinoids for the prophylaxis of CINV.

## Methods

A literature search was conducted in OVID Medline, EMBASE, and Cochrane Central Register of Controlled Trials from inception up until March 2024 (Appendix 1, Search strategy). Search strategy was designed with the concepts of cannabinoid (cannabis, dronabinol, cannabinoids, cannabidiol, nabilone, tetrahydrocannabinol), and chemotherapy-induced nausea, and vomiting. No restrictions were placed. Backwards reference screening of included studies was also conducted.

Articles were screened for eligibility by two reviewers (AB, JK) independently and in-duplicate after a screening exercise of ten articles. Articles were deemed eligible for further screening after level 1 title and abstract screening if they reported on patients with cancer, chemotherapy-induced nausea and vomiting, and cannabinoids. Articles were screened in level 2 full text and considered for data extraction if they reported on a randomized controlled trial with cannabinoids in one arm for the intent of prophylaxis.

Studies were ultimately included in this review and meta-analysis if they reported on one of four endpoints for our meta-analysis—complete response (no nausea and no vomiting, and no use of rescue medications), no nausea, no vomiting, or no use of rescue medication. Studies not reporting on any of these endpoints were excluded at the data extraction stage.

In data extraction, studies were reviewed and noted for study characteristics including study design (crossover vs. parallel groups design), inclusion criteria, exclusion criteria, measure of central tendency for age, and chemotherapy regimen. Antiemetic regimens were recorded as the differing medication between the treatment arms. Endpoints for meta-analysis were recorded in the overall phase if temporality was not specified. Where temporality was specified, data were recorded from the study by endpoint in either the acute (0–24-h post-chemotherapy), delayed (24–120-h post-chemotherapy), long-delayed (120 + h post-chemotherapy), or overall (0–120 h post-chemotherapy) phases. A composite endpoint was also produced, whereby the following data for each study was used to summarize the rate of CINV control in the following hierarchical order depending on availability: (i) complete response in overall phase, (ii) relative risk (RR) closer to the null hypothesis for complete response in either the acute or delayed phase, (iii) no nausea in the overall phase, (iv) lower RR for no nausea in either the acute or delayed phase, (v) no vomiting in the overall phase, and (vi) lower RR for no vomiting in either the acute or delayed phase.

A DerSimonian-Laird random effects model was used to generate summary effect estimates and corresponding 95% confidence intervals (CI) for the overall CINV control (composite endpoint) and each endpoint in the overall phase, and when there were more than one studies, in the acute, delayed, and long-delayed phases. Meta-analysis results are displayed graphically with forest plot. Subgroup analyses were conducted based on medication used in the control arm and study design. Additional analyses of cumulative meta-analysis and leave-one-out analysis were conducted for endpoints in the overall phase. Heterogeneity was assessed using *I*^2^ for endpoints with multiple studies, with values > 50% considered having notable heterogeneity. Publication bias was assessed using Egger’s test and represented graphically with funnel plots. Type I error was set at 0.05. StataBE 18.0 was used for all analyses.

For all included studies, quality assessment was conducted using the Cochrane Risk of Bias version 2 [16], and visualized using robvis [17].

## Results

The initial database search identified 871 records, and 17 further records were identified by backwards reference searching; after removal of 39 duplicates, a total of 888 records were screened. Ultimately, 26 studies [18–43] were included in this meta-analysis (Appendix Fig. 2 ).

Study demographics are presented in Table 1. Studies were published from 1975 to 2024, with 23 (88%) published before 2000. Of the studies, 18 were crossover, and 8 were parallel group studies. Three studies reported on pediatric patients and 23 reported on adult patients. Sample sizes ranged from 8 to 147. Chemotherapy regimens and antiemetic regimens are reported in depth in Table 1. Three studies reported on cannabinoid administered as levonatradol intramuscular injection, one study reported on cannabinoid (THC:CBD) administered as a spray, and the remaining 19 studies reported on cannabinoid administered in tablet form. The most commonly reported control arm regimens were placebo and prochlorperazine, together comprising fourth-fifths of studies included in this meta-analysis. Nearly half of published studies had some concern for bias (Appendix Figs. 3 and 4).

**Table 1:** Study characteristics

Study	Study design	Inclusion criteria	Exclusion criteria	Age (years)	Chemotherapy regimen	Cannabinoid	Comparator
**Sallan et al., 1975** [18]** (*n* = 22)**	Crossover	- Patients with neoplasm who had previously received cancer chemotherapeutic agents known to cause nausea and vomiting	- Pregnant women - Past history of emotional instability or untoward reactions to psychoactive drugs	Median = 29.5	Doxorubicin, 5-azacytidine, nitrogen mustard, imidazole carboxamide, procarbazine, high-dose cyclophosphamide, high-dose methotrexate Chemotherapy-naïve: 9%	Delta-9-tetrahydrocannabinol 15–20 mg PO Q4H three times	Placebo PO Q4H three times
**Chang et al., 1979 **[19] **(*n* = 15)**	Crossover	- Osteogenesis sarcoma who had undergone surgical removal of primary tumor and disease free upon entry into study - Receiving high-dose methotrexate therapy	- Patients likely to have untoward reactions to psychoactive drugs	Median = 24	High-dose methotrexate	Delta-9-tetrahydrocannabinol 10 mg/m^2^ PO Q3H five times, 1.93% THC one cigarette	Placebo capsules Q3H five times and one cigarette
**Ekert et al., 1979** [20] **(*n* = 19)** **Sponsor: R.P. Scherer Pty Ltd, Protea Pharmaceuticals Pty Ltd, Rotary Tableting Corporation** **Pty Ltd**	Crossover	- Patients requiring chemotherapy treatment	- Not reported	Median = 11	Methotrexate, doxorubicin, vincristine/doxorubicin/dacarbazine, vincristine/cyclophosphamide/doxorubicin/prednisolone, cytosine arabinoside/cyclophosphamide/asparaginase, cytosine arabinoside/6-thioguanine, 5-fluorouracil/doxorubicin/actinomycin d, vincirstine/lomustin	Delta 9 tetrahydrocannabinol 2.5–5.0 mg PO QID d1	Metoclopramide 5–10 mg PO QID d1
**Frytak et al., 1979 **[21]** (*n* = 116)**	Parallel groups	- Ambulatory outpatients with unresectable gastrointestinal cancer or participants in gastrointestinal cancer surgical adjuvant programs - Pretreatment oral intake of at least 1500 cal daily and could not have been experiencing nausea or vomiting before entry into the study - Undergoing initial chemotherapy exposure to combined 5-fluorouracil and semustine, either as two-drug combination or in three-drug combination with vincristine, doxorubicin, razoxane, or triazinate	- Taking psychotherapeutic agents or other antiemetics - Past history of drug dependence or significant psychological disturbance	Median = 61	5-fluorouracil and semustine, either as two-drug combination or in three-drug combination with vincristine, doxorubicin, razoxane or triazinate	Delta-9-tetrahydrocannabinol 15 mg PO TID d1-4	Prochlorperazine 10 mg PO TID or lactose PO TID d1-4
**Herman et al., 1979 **[22]** (*n* = 113) Sponsor: Eli Lilly**	Crossover	- Patients under treatment for repeated courses of chemotherapy, and had experienced severe drug-induced nausea and vomiting - No psychiatric or cardiovascular disease	- Not reported	Median = 33	Cisplatin/vinblastine/bleomycin, cyclophosphamide/doxorubicin/vincristine/prednisone, nitrogen mustard/vincristine/procarbazine/prednisone Chemotherapy-naïve: 0%	Nabilone 2 mg PO Q8H d1-5	Prochlorperazine 10 mg PO Q8H d1-5
**Orr et al., 1980 **[23]** (*n* = 55)**	Crossover	- Patients with neoplasms requiring drug therapy, with previously demonstrated repeated vomiting from anticancer agents known to induce emesis and have failed on standard antiemetic therapy	- Pregnant women - Receiving abdominal irradiation - Individuals with short life expectancy	Mean = 46	Doxorubicin, cyclophosphamide, doxorubicin/cyclophosphamide, doxorubicin/nitrosoureas, cyclophosphamide/ccnu, cyclophosphamide/5-fluorouracil/methotrexate, 5-fluouracil/methotrexate, ccnu, nitrogen mustard, cytosine arabinoside Chemotherapy-naïve: 0%	Delta-9-tetrahydrocannabinol 7 mg/m^2^ PO Q4H four times	Prochlorperazine 7 mg/m^2^ PO Q4H four times or placebo
**Steele et al., 1980 **[24]** (*n* = 37)**	Crossover	- Patients with cancer scheduled for chemotherapy	- Known cardiac disease or psychotic episodes or had regularly used marijuana	Not reported	High-dose Cis-di-chlorodiammineplatinum(II), low-dose DDP, mechlorethamine, streptozotocin, actinomycin D, DTIC	Nabilone 2 mg PO BID 3–5 times	Prochlorperazine 10 mg PO BID 3–5 times
**Chang et al., 1981 **[25]** (*n* = 8)**	Crossover	- Soft-tissue sarcoma who had undergone surgical removal of primary tumor and clinically disease free - Receiving adjuvant adriamycin and cyclophosphamide every 4 weeks	- Patients likely to have untoward reaction to psychoactive drugs	Median = 41	Doxorubicin/cyclophosphamide	Delta-9-tetrahydrocannabinol 10 mg/m^2^ PO Q3H five times, 1.93% THC one cigarette	Placebo capsules Q3H five times and one cigarette
**Neidhart et al., 1981 **[26]** (*n* = 52)**	Crossover	- Single injection or infusion of a cancer chemotherapeutic agent likely to induce intolerable vomiting - Experiencing incapacitating vomiting refractory to standard antiemetic agents with any prior cancer chemotherapy	- Not reported	Mean = 42.9	Cisplatin, doxorubicin, nitrogen mustard, cisplatin/doxorubicin	Delta-9-tetrahydrocannabinol 10 mg PO Q3-4H eight times	Haloperidol 2 mg PO Q3-4H eight times
**Johansson et al., 1982 **[27]** (*n* = 27)**	Crossover	- Patients receiving same cycles of cancer chemotherapy as previously, who had uncontrolled nausea and vomiting despite use of standard antiemetic drugs - Good performance status, ECOG < 2 - 1 day chemotherapy duration	- Known psychotic or cardiovascular diseases - Currently under medication (i.e., with phenothiazines) - Previous usage of marijuana	Not Reported	Cisplatin, doxorubicin, cyclophosphamide in combination with vinblastine/vincristine/ tegafur	Nabilone 2 mg PO BID four times	Prochlorperazine 10 mg PO BID four times
**Wada et al., 1982 **[28]** (*n* = 114) Sponsor: Eli Lilly**	Crossover	- Patients receiving cancer chemotherapy	- Significant cardiovascular, hepatic, renal or central nervous system diseases - Known psychosis or alcohol or drug addiction	Mean = 57	Doxorubicin, bischloroethylnitrosourea, bleomycin, cis-platinum, cyclophosphamide, dactinomycin, dtic, 5-fluorouracil, nitrogen mustard, melphalan, methotrexate, mitomycin, procarbazine, streptozotocin, tamoxifen, vinblastine, vincristine, VP-16	Nabilone 2 mg PO BID d1-5	Placebo PO BID d1-5
**Ahmedzai et al., 1983 **[29]** (*n* = 34) Sponsor: Eli Lilly**	Crossover	- 21-day cycles of combination chemotherapy comprising cyclophosphamide (CTX), adriamycin, etopside, vincristine - ECOG < = 3	- Not reported	Median = 58	Cyclophosphamide, adriamycin, VP-16, vincristine	Nabilone 1 mg PO BID d1-3	Prochlorperazine 10 mg PO TID d1-3
**Hutcheon et al., 1983 **[30]** (*n* = 108) Sponsor: Pfizer**	Parallel groups	- Inpatients scheduled to receive first course of potentially highly emetic cytotoxic chemotherapy	- Pregnancy - History of psychiatric disturbance or cardiovascular disease	Mean = 50.2	Cis-platinum, 5-fluorouracil/doxorubicin/mitomycin, cyclophosphamide/doxorubicin/vincristine, cyclophosphamide/doxorubicin, VP-16, cyclophosphamide/methotrexate/ 5-fluorouracil, mustine/ vinblastine/ procarbazine	Levonantradol 0.5–1.0 mg IM Q4H four times	Chlorpromazine 25 mg IM Q4H four times
**Sheidler et al., 1984 **[31]** (*n* = 16) Sponsor: Pfizer**	Crossover	- Patients with new and previously treated cancer admitted to receive chemotherapy	- Patients with brain metastases, neurologic impairment, severe cardiac, renal or hepatic disease, or history of emotional instability - Pregnant women - On psychoactive drugs	Not reported	Cisplatin, cyclophosphamide, doxorubicin	Levonantradol 1 mg IM Q4H four times	Prochlorperazine 10 mg IM Q4H four times
**Stambaugh et al., 1984 **[32]** (*n* = 20) Sponsor: Pfizer**	Parallel groups	- Persistent nausea and vomiting from cancer chemotherapy determined to be refractory to maximally recommended doses of conventional antiemetics	- Severe liver disease - Severe renal disease - CNS metastasis	Not reported	Not reported	Levonantradol 0.5, 1.0, 1.5, 2.0 mg IM Q4H four times	Placebo IM Q4H four times
**Niiranen et al., 1985 **[33]** (*n* = 24) Sponsor: Eli Lilly**	Crossover	- Patients with lung cancer who had been listed for treatment with at least two identical consecutive cycles of chemotherapy	- Clinically significant hepatic, renal, or central nervous system disease – Alcoholism - Drug addiction	Mean = 61	Cyclophosphamide, VP-16, vincristine, doxorubicin, cisplatin, vindesine Chemotherapy-naïve: 42%	Nabilone 1 mg PO BID d1-5	Prochlorperazine 7.5 mg PO BID d1-5
**Dalzell et al., 1986 **[34]** (*n* = 23) Sponsor: Eli Lilly**	Crossover	- Scheduled to receive two identical courses of antineoplastic chemotherapy for malignant disease	- Not reported	Median = 7	Vincristine/actinomycin/cyclophosphamide, cisplatinum/vp-16, mustine/vincristine/procarbazine/ prednisolone, methotrexate ammonium methylsulfonate/vp-16/5-azacytidine, high dose cytarabine, vincristine/cyclophosphamide/cisplatinum, daunorubican/cytarabine/ thioguanine	Nabilone 0.5 mg-1 mg PO BID-TID d1	Domperidone 5–15 mg PO BID d1
**Niederle et al., 1986 **[35]** (*n* = 20)**	Crossover	- Patients with testicular cancer	- Not reported	Median = 25	Cisplatin/doxorubicin	Nabilone 2 mg PO BID d1-5	Alizapride 150 mg PO TID d1-5
**Chan et al., 1987 **[36]** (*n* = 30) Sponsor: Eli Lilly**	Crossover	- Repeated courses of chemotherapy - Experienced severe drug-induced nausea and vomiting, but have never received either nabilone or prochlorperazine previously	- Cis-platinum-based regimens	Mean = 11.8	Vincristine, doxorubicin, cyclophoshamide, teniposide, mechlorethamine/ vincristine/ procarbazine/ prednisone, cytosine arabinoside, methotrexate, doxorubicin/ dactinomycin/ vincristine/ cyclophosphamide, 5-fluorouracil, prednisone, bleomycin/ cyclophosphamide/ dactinomycin, adriamycin/ bleomycin/ vinblastine/ dacarbazine, etoposide, 6-thioguanine Chemotherapy-naïve: 0%	Nabilone 0.5–1–2 mg BID-TID (depending on weight) d1-3	Prochlorperazine 2.5–10 mg BID-TID (dependent on weight) d1-3
**Cunningham et al., 1988 **[37]** (*n* = 80) Sponsor: Eli Lilly**	Crossover	- Histologically confirmed cancer and receiving first course of cisplatin 20–50 mg/m^2^	- History of psychiatric illness - Past history of extrapyramidal reactions	Mean = 42	Cisplatin	Nabilone 2 mg PO BID four-five times + prochlorperazine 5 mg PO BID four doses	Metoclopramide 2 mg/kg loading dose and 3 mg/kg IV infusion over 8 h + dexamethasone 20 mg IV once
**McCabe et al., 1988 **[38]** (*n* = 36)**	Crossover	- Patients undergoing chemotherapeutic treatment, no prior history of psychiatric illness or pre-existing cardiac disease - Performance status 0–1 - Experienced severe nausea and vomiting that was refractory to standard antiemetics	- Not reported	Median = 46	Doxorubicin, cyclophosphamide/methotrexate/5-fluorouracil, nitrogen mustard/ vincristine/procarbzine/prednisone, platinum combinations, dtic, 5-fluorouracil combinations, 5-azacytadine Chemotherapy-naïve: 0%	Delta-9-tetrahydrocannabinol 15 mg/m^2^ PO Q4H d1	Prochlorperazine 10 mg PO Q4H d1
**Lane et al., 1990 **[39]** (*n* = 19) Sponsor: Roxanne Laboratories and UNIMED**	Parallel groups	- Biopsy-proven cancer, who had completed at least one course of chemotherapy within 6 months prior to enrolment and scheduled for treatment with standard chemotherapy other than high-dose cisplatin - KPS > 60% - Serum creatinine below 2.0 mg/dl - Bilirubin less than 1.5 mg/dl - Leukocyte count > 4000/mm^3^ - Platelet count greater than 100,000/mm^3^	- Not reported	Median = 52	Bleomycin, 5-fluorouracil, methotrexate, vinblastine, vincristine, bischloroethylnitrosourea, cyclophosphamide, decarbazine, doxorubicin, etoposide, mitomycin	Dronabinol 10 mg PO Q6H d1-5	Prochlorperazine 10 mg PO Q6H d1-5
**Lane et al., 1991 **[40]** (*n* = 62) Sponsor: Roxanne Laboratories and UNIMED**	Parallel groups	- Patients being treated for cancer with chemotherapy other than investigational agents or high-dose (> 60 mg/m^2^) cisplatin - KPS > 60% - Serum creatinine below 2.0 mg/dl - Bilirubin less than 1.5 mg/dl - Leukocyte count greater than 4000/mm^3^ - Platelet count greater than 100,000/mm^3^	- Central nervous system primaries or metastases	Median = 55	Bleomycin, cyclophosphamide, fluouracil, methotrexate, vinblastine, vincristine Chemotherapy-naïve: 0%	Dronabinol 10 mg PO QID d1-6	Prochlorperazine 10 mg PO QID d1-6
**Meiri et al., 2007 **[41]** (*n* = 64) Sponsor: Solvay Pharmaceuticals**	Parallel groups	- Patients with malignancy that did not involve bone marrow and be undergoing chemotherapy including a moderately to highly emetogenic regimen, oxaliplatin at doses employed for treatment of colon cancer, or combination of doxorubicin with cyclophosphamide (600 mg/m^2^) with or without taxanes for the treatment of breast cancer - Life expectancy of at least 6 weeks postchemotherapy - ECOG 0–2	- History of anticipatory nausea and/or vomiting - Primary malignancy of brain, spinal cord of nervous system; metastases to these sites; leukemias or lymphomas involving the bone marrow - History of brain surgery, moderate to severe brain trauma or any other neurological disorder likely to affect central nervous system functioning - Marijuana use within 30 days of baseline and antiemetic agents, including diphenhydramine, within 7 days before baseline - Patients prescribed opiates, propoxyphene or benzodiazepines by treating physician whose dosage was not stable for 2 weeks before study entry	Mean + / − SD = 57.9 + / − 12.0	Moderately to highly emetogenic regimen, oxaliplatin at doses employed for treatment of colon cancer, or combination of doxorubicin with cyclophosphamide (600 mg/m^2^) with or without taxanes for the treatment of breast cancer Chemotherapy-naïve: 0%	Dronabinol 2.5 mg PO BID d1 and QID d2-5	Placebo
**Duran et al., 2010 **[42]** (*n* = 16)**	Parallel groups	- Karnofsky score > = 70 with CINV lasting more than 24 h despite prophylaxis with standard anti-emetic treatment after administration of 1-day moderately emetogenic chemotherapy during previous chemotherapy cycle - Histologically confirmed solid tumors	- Current use of illicit drugs, THC or alcohol abuse confirmed by rapid urine screen - Abnormal laboratory values: WBC < 3000 mm^3^, platelet count < 100,000/mm^3^, AST > 2.5 × upper limit of normal, ALT > 2.5 × upper limit of normal, creatinine > 1.5 mg/dl - Multiple-day chemotherapy in a single cycle - Radiation therapy on the abdomen or pelvis within 1 week before or during the study - Cannabinoid use within 30 days prior to enrolment - History of major psychiatric disorder, severe cardiovascular disease, seizures, were pregnancy or lactating, or had suspected hypersensitivity to cannabinoids	Median = 50	Carboplatin, Cisplatin < = 50 mg/m^2^, Cyclophosphamide < 1500 mg/m^2^, Doxorubicin > 60 mg/m^2^, Idarubicin, Ifosfamide, Irinotecan, Mitoxantrone < 15 mg/m^2^, Epirubicin < 90 mg/m^2^	Delta-9-tetrahydrocannabinol 2.7 mg and cannabidiol 2.5 mg spray −2 h, 30 min and 120 min after chemotherapy	Placebo 2.5 mg spray −2 h, 30 min and 120 min after chemotherapy
**Grimison et al., 2024 **[43]** (*n* = 147) Sponsor: Tilray Brands**	Parallel groups	- Solid tumor or hematologic malignancy of any stage, being treated with intravenous chemotherapy of moderate or high emetogenic risk, and scheduled for at least two more consecutive cycles of the same chemotherapy- Experienced refractory nausea and vomiting in earlier treatment cycle of same chemotherapy, despite guideline-consistent antiemetic prophylaxis	- ECOG > 2 - Contraindication to medicinal cannabis such as unstable cardiovascular disease, substance use disorder, or significant mental health disorder - Experiencing disease-related nausea and vomiting - Receiving concomitant oral chemotherapy - Radiotherapy to the brain or gastrointestinal tract during the study period	Median = 56	Anthracycline, Carboplatin, FOLFOX/ Biological, Cisplatin, FOLFIRINOX	Oral THC:CBD 2.5–10 mg PO TID d1-5	Placebo 2.5–10 mg PO TID d1-5

### Overall CINV control

**Fig. 1:**
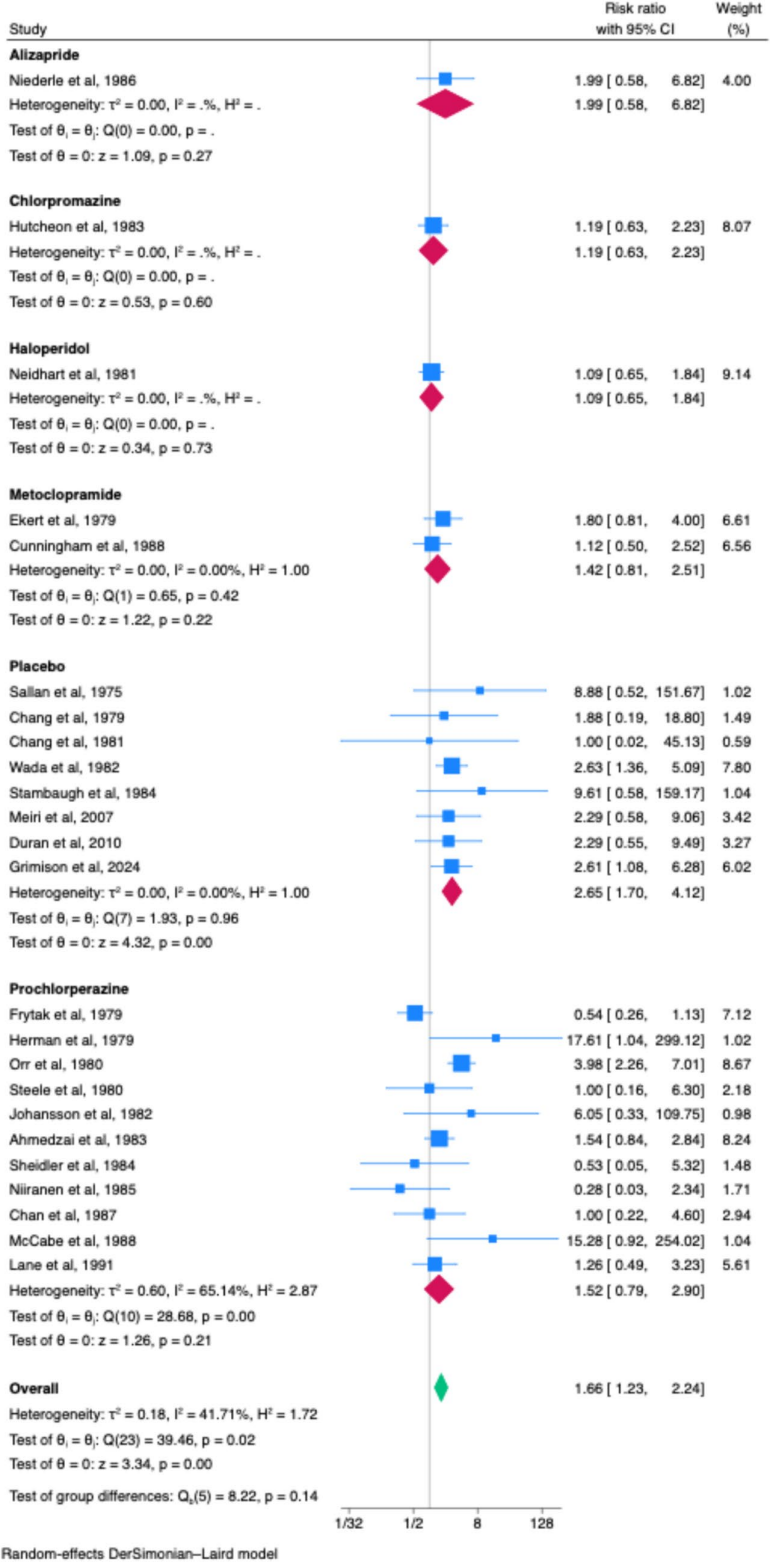
Overall CINV control (composite endpoint)

Twenty-four studies reported on overall CINV control, reporting on either “complete response” (no vomiting, retching, or use of rescue medication), or no nausea or no vomiting. Cannabinoid had superior overall CINV control compared to placebo (RR 2.65, 95% CI 1.70–4.12, *n* = 8, *I*^2^ = 0.00%). There was no difference between cannabinoid and active treatment alternatives of alizapride (RR 1.99, 95% CI 0.58–6.82. *n* = 1), chlorpromazine (RR 1.19, 95% CI 0.63–2.23, *n* = 1), haloperidol (RR 1.09, 95% CI 0.65–1.84, *n* = 1), metoclopramide (RR 1.42, 95% CI 0.81–2.51, *n* = 2, *I*^2^ = 0.00%), and prochlorperazine (RR 1.52, 95% CI 0.79–2.90, *n* = 11, *I*^2^ = 65.14%) (Fig. 1).

In the cumulative meta-analysis, the summary effect estimate remained stable over publication history (Appendix Fig. 5). Leave-one-out analysis identified no single substantial influential study (Appendix Fig. 6). There was no difference in effect estimate by study design (*p* = 0.93, Appendix Fig. 7). There was no concern for publication bias (*p* = 0.244, Appendix Fig. 8).

### Complete response

Fifteen studies reported on complete response: 11 in the overall phase, 4 in the acute phase, and 3 in the delayed phase. In the overall phase, cannabinoid had superior complete response compared to placebo (RR 2.51, 95% CI 1.55–4.05, *n* = 5, *I*^2^ = 0.00%, Appendix Fig. 9). There was no difference between cannabinoid and active treatment alternatives of metoclopramide (RR 1.12, 95% CI 0.50–2.52, *n* = 1) and prochlorperazine (RR 1.86, 95% CI 0.64–5.40, *n* = 5, *I*^2^ = 40.34%) (Appendix Fig. 9). In the acute phase, there was no difference between cannabinoid and placebo (RR 2.09, 95% CI 0.28–15.80, *n* = 2, *I*^2^ = 54.05%), haloperidol (RR 1.09, 95% CI 0.65–1.84,* n* = 1) and prochlorperazine (RR 0.54, 95% CI 0.26–1.13, *n* = 1) (Appendix Fig. 10). In the delayed phase, there was no difference between cannabinoid and placebo (RR 2.29, 95% CI 0.85–6.15, *n* = 2, *I*^2^ = 0.00%), and prochlorperazine (RR 0.89, 95% CI 0.57–1.38, *n* = 1) (Appendix Fig. 11).

In the cumulative meta-analysis, the summary effect estimate remained stable over publication history (Appendix Fig. 12). Leave-one-out analysis identified no single substantial influential study (Appendix Fig. 13). There was no difference in effect estimate by study design (*p* = 0.92, Appendix Fig. 14). There was no concern for publication bias (*p* = 0.636, Appendix Fig. 15).

### No nausea

Fourteen studies reported on nausea control: 10 in the overall phase, 3 in the acute phase, and 4 in the delayed phase. In the overall phase, there was no difference between cannabinoid and placebo (RR 1.59, 95% CI 0.22–11.40, *n* = 2, *I*^2^ = 0.00%), chlorpromazine (RR 1.19, 95% CI 0.63–2.23, *n* = 1), metoclopramide (RR 1.07, 95% CI 0.44–2.64, *n* = 2, *I*^2^ = 72.24%), and prochlorperazine (RR 1.58, 95% CI 0.55–4.52, *n* = 5, *I*^2^ = 59.27%) (Appendix Fig. 16). In the acute phase, there was no difference between cannabinoid and alizapride (RR 1.99, 95% CI 0.58–6.82, *n* = 1), and prochlorperazine (RR 0.93, 95% CI 0.33–2.60, *n* = 2, *I*^2^ = 78.24%) (Appendix Fig. 17). In the delayed phase, cannabinoids were superior to alizapride (RR 3.41, 95% CI 1.12–10.41, *n* = 1). There was no difference between cannabinoid and placebo (RR 2.00, 95% CI 0.46–8.76, *n* = 1), and prochlorperazine (RR 1.14, 95% CI 0.66–1.99, *n* = 2, *I*^2^ = 57.46%) (Appendix Fig. 18).

In the cumulative meta-analysis, the summary effect estimate remained stable over publication history (Appendix Fig. 19). Leave-one-out analysis identified no single substantial influential study (Appendix Fig. 20). There was no difference in effect estimate by study design (*p* = 0.62, Appendix Fig. 21). There was no concern for publication bias (*p* = 0.820, Appendix Fig. 22).

### No vomiting

Twelve studies reported on vomiting control: 8 in the overall phase, 3 in the acute phase, and 4 in the delayed phase. There was no difference between cannabinoid and placebo (RR 1.19, 95% CI 0.87–1.62, *n* = 4, *I*^2^ = 0.00%), chlorpromazine (RR 1.21, 95% CI 0.70–2.11, *n* = 1), metoclopramide (RR 2.47, 95% CI 0.09–67.65, *n* = 2, *I*^2^ = 82.22%), and prochlorperazine (RR 1.00, 95% CI 0.27–3.69, *n* = 1) (Appendix Fig. 23). In the acute phase, there was no difference between cannabinoid and alizapride (RR 2.20, 95% CI 0.47–10.23, *n* = 1), and prochlorperazine (RR 1.03, 95% CI 0.72–1.48, *n* = 2, *I*^2^ = 0.00%) (Appendix Fig. 24). In the delayed phase, cannabinoids were superior to alizapride (RR 3.41, 95% CI 1.12–10.41, *n* = 1). There was no difference between cannabinoid and placebo (RR 2.00, 95% CI 0.46–8.76, *n* = 1) and prochlorperazine (RR 1.14, 95% CI 0.66–1.99, *n* = 2, *I*^2^ = 57.46%) (Appendix Fig. 25).

In the cumulative meta-analysis, the summary effect estimate trended towards reporting cannabinoid as equivalent to control arms over publication history (Appendix Fig. 26). Leave-one-out analysis identified no single substantial influential study (Appendix Fig. 27). There was no difference in effect estimate by study design (*p* = 0.36, Appendix Fig. 28). There was concern for publication bias (*p* = 0.042, Appendix Fig. 29).

### No use of rescue medications

Four studies reported on the use of rescue medications in the overall phase. Cannabinoids led to no difference in use of rescue medication compared to placebo, (RR 1.69, 95% CI 0.81–3.54, *n* = 2, *I*^2^ = 49.07%), domperidone (RR 1.36, 95% CI 0.67–2.77, *n* = 1), and prochlorperazine (RR 1.08, 95% CI 0.73–1.59, *n* = 1) (Appendix Fig. 30).

In the cumulative meta-analysis, the summary effect estimate trended towards reporting cannabinoid as having lower rates of use of rescue medications over publication time (Appendix Fig. 31). Leave-one-out analysis identified no single substantial influential study (Appendix Fig. 32). There was no difference in effect estimate by study design (*p* = 0.91, Appendix Fig. 33). There was no concern for publication bias (*p* = 0.140, Appendix Fig. 34).

## Discussion

In this meta-analysis, we report that cannabinoids are superior to placebo for overall control of CINV, but no better than alizapride, chlorpromazine, dexamethasone, and prochlorperazine. However, the majority of these studies were published before 2000 and therefore not using modern antiemetic regimens as comparators; most of the older trials comparing cannabinoids to placebo used cannabinoids are monotherapy prophylaxis and used THC without CBD. In a recent phase II–III study by Grimison et al. [43, 44] where THC:CBD was added as an adjunct to existing guideline-concordant antiemetic prophylaxis for patients scheduled to receive moderately or highly emetogenic chemotherapy and who had refractory nausea and vomiting in a previous chemotherapy cycle, there were promising results reporting that adjunctive cannabis can improve complete control rate.

There has been longstanding interest in cannabinoids for treatment of CINV over the past 5 decades, but no definitive evidence for their efficacy compared to monotherapy antiemetics [14]. As well, given the lack of trials in the modern era of antiemetics and the variability of cannabinoids reported in the literature, it is unlikely cannabinoids will gain increasing global favor by guideline committee without further trials. The recent trial by Grimison et al. [43, 44] is promising, but further trials in the modern era of antiemetics are needed to better evaluate efficacy. Future trials should also investigate whether certain types of cannabinoids (either THC alone, THC:CBD combination) are more efficacious than others.

Of note, there are existing concerns regarding adverse effects for cannabinoids, with many studies reporting impaired short-term memory, impaired motor coordination, paranoia, psychosis, and sedation [45, 46]. Adverse effects can be managed with slow titration and careful monitoring. Nevertheless, consideration for use and further study of cannabinoids should consider safety concerns given the well-documented adverse effects that cannabinoids can cause. Should cannabinoids be considered further for prophylaxis of CINV, further studies should investigate management of adverse effects as well.

This meta-analysis has several strengths compared to prior meta-analyses [15, 47]. It focused specifically on efficacy, had increased statistical power with the inclusion of modern studies, and included analysis of a composite endpoint for overall CINV control. Furthermore, cumulative and leave-one-out meta-analyses were conducted, for which there were no single influential studies noted.

There are also multiple limitations. As is inherent in systematic review methodology, the strength of the meta-analysis conclusion is dependent on the quality of underlying studies. There was some concern for bias in nearly half of the studies. Most studies were reported before the era of modern antiemetics and therefore not applicable currently. Furthermore, there was heterogeneity of endpoints across older studies, for which we made all reasonable efforts to extract data into standardized endpoints. There was also heterogeneity of tumor population and emetogenicity of chemotherapies within and across studies and route of administration (notably, there is no trial on the inhaled form of cannabinoids). There should therefore be careful interpretation of these results, with emphasis placed on the recent study by Grimison et al. [43, 44] which suggests that THC:CBD as an adjunct to modern standard antiemetic regimens (albeit mostly without olanzapine) may lead to improved efficacy. Finally, this meta-analysis did not focus on safety; safety has been extensively reported in other studies.

In conclusion, there is a paucity of studies of cannabinoids in the context of modern triple or quadruple therapy treatment regimens for CINV. One study suggests that THC:CBD may have efficacy for secondary prevention of refractory CINV. Further studies should further investigate efficacy, particularly compared to olanzapine-containing regimens, and also evaluate safety. In middle to higher income countries, should cannabinoids’ efficacy continue to be documented, further work can be focused on determining whether certain subgroups of populations (i.e., patients receiving moderately and/or highly emetogenic chemotherapy) tend to experience greater efficacy, as it may be more cost-effective than expensive antiemetics.

## Data Availability

No datasets were generated or analysed during the current study.

## Appendices

Appendix 1. Search strategy

Appendix 2. Figure 2 PRISMA diagram

Appendix 3. Quality assessment

3.1. Fig. 3 Summary

3.2. Figure 4 By study.

Appendix 4. Composite endpoint – additional analyses

4.1. Figure 5 Cumulative meta-analysis.

4.2. Figure 6 Leave-one-out analysis.

4.3. Figure 7 By study design.

4.4. Figure 8 Funnel plot.

Appendix 5. Complete response

5.1. Figure 9 Overall phase.

5.2. Figure 10 Acute phase.

5.3. Figure 11 Delayed phase.

Appendix 6. Complete response – additional analyses

6.1. Figure 12 Cumulative meta-analysis.

6.2. Figure 13 Leave-one-out analysis.

6.3. Figure 14 By study design.

6.4. Figure 15 Funnel plot.

Appendix 7. No nausea

7.1. Figure 16 Overall phase.

7.2. Figure 17 Acute phase.

7.3. Figure 18 Delayed phase.

Appendix 8. No nausea – additional analyses

8.1. Figure 19 Cumulative meta-analysis.

8.2. Figure 20 Leave-one-out analysis.

8.3. Figure 21 By study design.

8.4. Figure 22 Funnel plot.

Appendix 9. No vomiting

9.1. Figure 23 Overall phase.

9.2. Figure 24 Acute phase.

9.3. Figure 25 Delayed phase.

Appendix 10. No vomiting – additional analyses

10.1. Figure 26 Cumulative meta-analysis.

10.2. Figure 27 Leave-one-out analysis.

10.3. Figure 28 By study design.

10.4. Figure 29 Funnel plot.

Appendix 11. No use of rescue medications

11.1. Figure 30 Overall phase.

11.2. Figure 31 Cumulative meta-analysis.

11.3. Figure 32 Leave-one-out analysis.

11.4. Figure 33 By study design.

11.5. Figure 34 Funnel plot.
